# Expert consensus on re-irradiation for recurrent glioma

**DOI:** 10.1186/s13014-017-0928-3

**Published:** 2017-12-01

**Authors:** Andra Krauze, Albert Attia, Steve Braunstein, Michael Chan, Stephanie Combs, Rainer Fietkau, John Fiveash, John Flickinger, Anca Grosu, Steven Howard, Carsten Nieder, Maximilian Niyazi, Lindsay Rowe, Dee Dee Smart, Christina Tsien, Kevin Camphausen

**Affiliations:** 1 Radiation Oncology Branch, National Cancer Institute NIH, Bethesda, MD USA; 2 Department of Radiation Oncology, Vanderbilt University Medical Center, Nashville, TN USA; 3 Department of Radiation Oncology, University of California, San Francisco, USA; 4 Department of Radiation Oncology, Wake Forest Baptist Medical Center, Winston-Salem, NC USA; 5 Department of Radiation Oncology, Technical University of Munich (TUM), Munich, Germany; 6 Institute of Innovative Radiotherapy (iRT), Department of Radiation Sciences (DRS), Helmholtz Zentrum München, Neuherberg, Germany; 7 Deutsches Konsortium für Translationale Krebsforschung (DKTK), Partner Size, Munich, Germany; 8 Department of Radiotherapy Erlangen, Erlangen, Germany; 9 Department of Radiation Oncology, University of Alabama at Birmingham, Birmingham, AL USA; 10 UPMC Presbyterian-Shadyside, Pittsburgh, PA USA; 11 Department of Radiation Oncology, University of Freiburg, Freiburg, Germany; 12 Department of Human Oncology, University of Wisconsin, Madison, WI USA; 13 Department of Oncology and Palliative Medicine, Hospital Trust, 8092 Bodø, Nordland Norway; 14 Department of Clinical Medicine, Faculty of Health Sciences, University of Tromsø, 9038 Tromsø, Norway; 15 Department of Radiation Oncology, LMU Munich, Marchioninistr. 15, 81377 Munich, Germany; 16 German Cancer Consortium (DKTK), German Cancer Research Center (DKFZ), Heidelberg, Germany; 17 Radiation Oncology Branch, National Cancer Institute, NIH, Bethesda, MD USA; 18 Department of Radiation Oncology, Washington University, St. Louis, MO USA

## Abstract

**Purpose:**

To investigate radiation oncologists’ opinions on important considerations to offering re-irradiation (re-RT) as a treatment option for recurrent glioma.

**Materials and methods:**

A survey was conducted with 13 radiation oncologists involved in the care of central nervous system tumor patients. The survey was comprised of 49 questions divided into 2 domains: a demographic section (10 questions) and a case section (5 re-RT cases with 5 to 6 questions representing one or several re-RT treatment dilemmas as may be encountered in the clinic). Respondents were asked to rate the relevance of various factors to offering re-RT, respond to the cases with a decision to offer re-RT vs. not, volume to be treated, margins to be employed, dose/fractionation suggested and any additional comments with respect to rationale in each scenario.

**Results:**

Sixty nine percent of responders have been practicing for greater than 10 years and 61% have re-RT 20 to 100 patients to date, with 54% seeing 2–5 re-RT cases per month and retreating 1–2 patients per month. Recurrent tumor volume, time since previous radiation therapy, previously administered dose to organs at risk and patient performance status were rated by the majority of responders (85%, 92%, 77%, and 69% respectively) as extremely relevant or very relevant to offering re-RT as an option.

**Conclusion:**

The experts’ practice of re-RT is still heterogeneous, reflecting the paucity of high-quality prospective data available for decision-making. Nevertheless, practicing radiation oncologists can support own decisions by referring to the cases found suitable for re-RT in this survey.

**Electronic supplementary material:**

The online version of this article (10.1186/s13014-017-0928-3) contains supplementary material, which is available to authorized users.

## Introduction

Seventy-eight thousand brain tumors are diagnosed in the United States per year with gliomas representing approximately one third [1]. Virtually all gliomas eventually recur following treatment and in their natural history carry devastating neurological and psychological implications for those affected. Patients with a diagnosis of glioma may undergo multiple resections, radiation therapy and multiple lines of systemic treatment with diminishing treatment options as the disease progresses. Re-irradiation (re-RT) as a possible treatment option often enters the discussion and multiple retrospective studies have shown re-RT to be feasible and to improve outcome in selected patients [2–13]. The benefits of re-RT include possible palliation by way of decreased steroid use, improvement in neurological symptoms, and in some patients an improvement in progression free survival and possibly overall survival. The timing of re-RT offering the best opportunity for benefit is unclear. Patient selection for re-RT varies according to the study, institution and available techniques and hence the type of patient who may benefit from its administration remains unclear. Prognostic scores using retrospective data have attempted to identify patient strata that may benefit from re-RT based on patient and histological factors [14–17]. Significant heterogeneity in management has proven difficult with some studies reporting validation of existing scoring systems and others not. The radiation dose to be administered and its fractionation as well as the co-administration of chemotherapy suffer from lack of consensus and are as yet unclear. Re-RT is the subject of several ongoing trials, the results thereof, although highly relevant and anticipated, nonetheless are likely to leave us with unanswered questions. Many patients with recurrent glioma who may be considered for re-RT in the clinic would not necessarily have been eligible for the ongoing trials but according to existing data may nonetheless derive benefit from re-RT and may receive re-RT when no other options are available.

The intent of this publication was to obtain the opinion of radiation oncologists who 1) share an interest in the re-RT of patients with recurrent glioma defined as having published at least one paper on the subject and or having an ongoing practice or open protocol pertaining to re-RT or 2) have treated a significant number of cases. A simple but robust survey was employed as the springboard for expert discussion in an effort to gain a better understanding of patient selection and the philosophy underlying the re-RT dose and fractionation being offered.

## Materials and methods

### Survey development

The survey began with the referral and treatment of patients with recurrent glioma seen in clinic and presented at NCI NIH CNS tumor boards where re-RT as option came up and a radiation oncology opinion was requested. We noted that cases shared features that made the decision to offer re-RT or the feasibility thereof from a planning perspective difficult. Knowing that the level of expertise of the responder and the clinical load of re-RT cases they are faced with, was as relevant as the cases themselves, the survey is divided into 2 domains: a demographic section containing 10 questions aiming to assess the load of re-RT cases the responder has treated/is treating and their institution of practice and a second section containing 5 re-RT cases with 5 to 6 questions associated with each case aimed at eliciting a response with respect to one or several re-RT treatment dilemmas as may be encountered in the clinic. The survey consisted of 49 total questions over 7 pages with 6 associated images (Additional file 1).

### Question generation

The Survey Monkey survey (Additional file 1) was developed in several stages starting with review of the literature in conjunction with commonly arising re-RT questions in the clinic. This was followed by multiple iterations of the questionnaire itself which was written by the primary author (AK), and reviewed by two experts (KC and DS) for relevance, accuracy and usability of each item. Subsequently the survey was tested by KC, DS and LR for functionality. With respect to factors of relevance to offering re-RT as a treatment option clinically identified and literature described factors were included. However, we also gave the responder the opportunity to add other factors they felt were relevant in addition to the ones provided.

### Survey distribution

The survey was distributed with the intent of obtaining relevant responses from radiation oncologists with a range of experience who share an interest in re-RT of patients with recurrent glioma as evidenced by previous publications on the subject and/or upon recommendation of a peer who shared that interest. In order to capture the wide range of approaches to this challenging clinical scenario, we did elect to also include responders who are just starting out in re-irradiation and “up and coming” investigators with an interest in the field. The ultimate goal is to have a wider range of responders post publication when the survey is open to readers. The 13 potential responders were contacted by email and informed of the research proposed in association with the survey and offered contribution to the publication and all responded to the questionnaire.

The responders were asked to provide both binary answers regarding the intention to re-irradiate as well as quantitative answers with respect to margins and doses employed and were given the option to explain their rationale in an associated comment section. The questionnaire was administered between August and November 2016.

## Results

### Survey results

Thirteen radiation oncology experts, 8 from the United States and 5 from Europe responded to the survey and are included as co-authors on this manuscript. All of the responses have been anonymized.

#### Expertise of responders

**Fig. 1:**
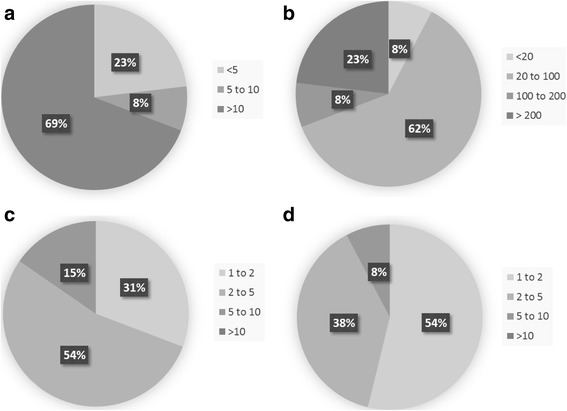
Level of expertise and experience of survey responders. **a** Number of years practicing radiation oncology. **b** Number of patients re-irradiated to date. **c** Number of potential glioma re-irradiation cases seen per month. **d** Number of patients re-irradiated per month

Sixty nine percent (9/13) of the contributing radiation oncologists have been practicing for greater than 10 years (Fig 1). The majority, 62% (8/13) have re-irradiated 20 to 100 patients to date and 54% (7/13) see 2 to 5 potential re-RT cases per month. Fifty four percent (7/13) retreat 1–2 patients per month and 38% (5/13) retreat 2 to 5 patients. For 85% (11/13) of responders “some of the patients” receive re-RT on a clinical research protocol.

#### Factors underlying the decision to offer re-RT

Responders were offered 11 clinical factors to rate in terms of relevance to the decision to offer re-RT (Table 1). Time since previous radiation therapy, tumor volume, previously administered dose to organs at risk and patient performance status were felt by the majority of responders (92%, 85%, 77%, and 69% respectively) to be extremely relevant or very relevant to offering re-RT as an option (Table 1). This was followed by patient age, number of lines of previous treatment, original histology, and previous use of bevacizumab (BEV)/BEV failure which were rated as very relevant or somewhat relevant in 93%, 84%, 77%, 77% respectively. No responders rated patient age, number of lines of previous treatment, DWI perfusion characteristics and FDG-PET avidity as extremely relevant but 1 responder (a different one in each case) did rate original histology, tissue documentation of recurrence, and previous use of BEV/BEV failure as extremely relevant, conversely. Fifty four percent of responders rated available tissue documentation of tumor progression and DWI characteristics as somewhat relevant, 46% previous use of BEV/BEV failure as somewhat relevant while 46% rated PET avidity of the lesion as not at all relevant. Responders were given the opportunity to comment on additional factors they would consider relevant when considering offering re-RT as free text in the survey. Responses included rate of tumor growth, molecular markers (IDH mutation status, MGMT methylation status), availability of high precision techniques, patient preference, favorable location vs. overlapping OAR, long natural history, surgical resectability and unifocal vs. multifocal disease and ability to travel for treatment. The presence of symptoms was not noted as an additional factor by any of the responders. In assessing which measures are being routinely performed as part of decision making when re-RT is considered 100% of the responders employed multidisciplinary discussions, 100% obtained previous RT summaries including DVH and field arrangements, nearly 92% also obtained the plan in DICOM format to create a plan sum and 100% calculated cumulative dose to OAR taking into account the previous treatment plans.

**Table 1:** Factors considered in offering re-irradiation as a treatment option

	Responder number/(%)			
Factor	Extremely relevant	Very relevant	Somewhat relevant	Not at all relevant
Tumor volume	5 (38)	7 (54)	1 (8)	0 (0)
Time since previous radiation therapy	6 (46)	6 (46)	1 (8)	0 (0)
Dose previously administered to organs at risk in the field	6 (46)	4 (31)	3 (23)	0 (0)
Patient performance status	5 (38)	4 (31)	4 (31)	0 (0)
Patient age	0 (0)	8 (62)	4 (31)	1 (8)
Original histology	1 (8)	4 (31)	6 (46)	2 (15)
Number of lines of previous treatment	0 (0)	5 (38)	6 (46)	2 (15)
Previous use of Bevacizumab or Bevacizumab failure	1 (8)	4 (31)	6 (46)	2 (15)
Available tissue documentation of tumor progression	1 (8)	3 (23)	7 (54)	2 (15)
Perfusion characteristics on diffusion weighted imaging (DWI)	0 (0)	6 (46)	7 (54)	0 (0)
PET avidity of the lesion	0 (0)	4 (31)	3 (23)	6 (46)

### Re-irradiation cases

#### Case 1

**Fig. 2:**
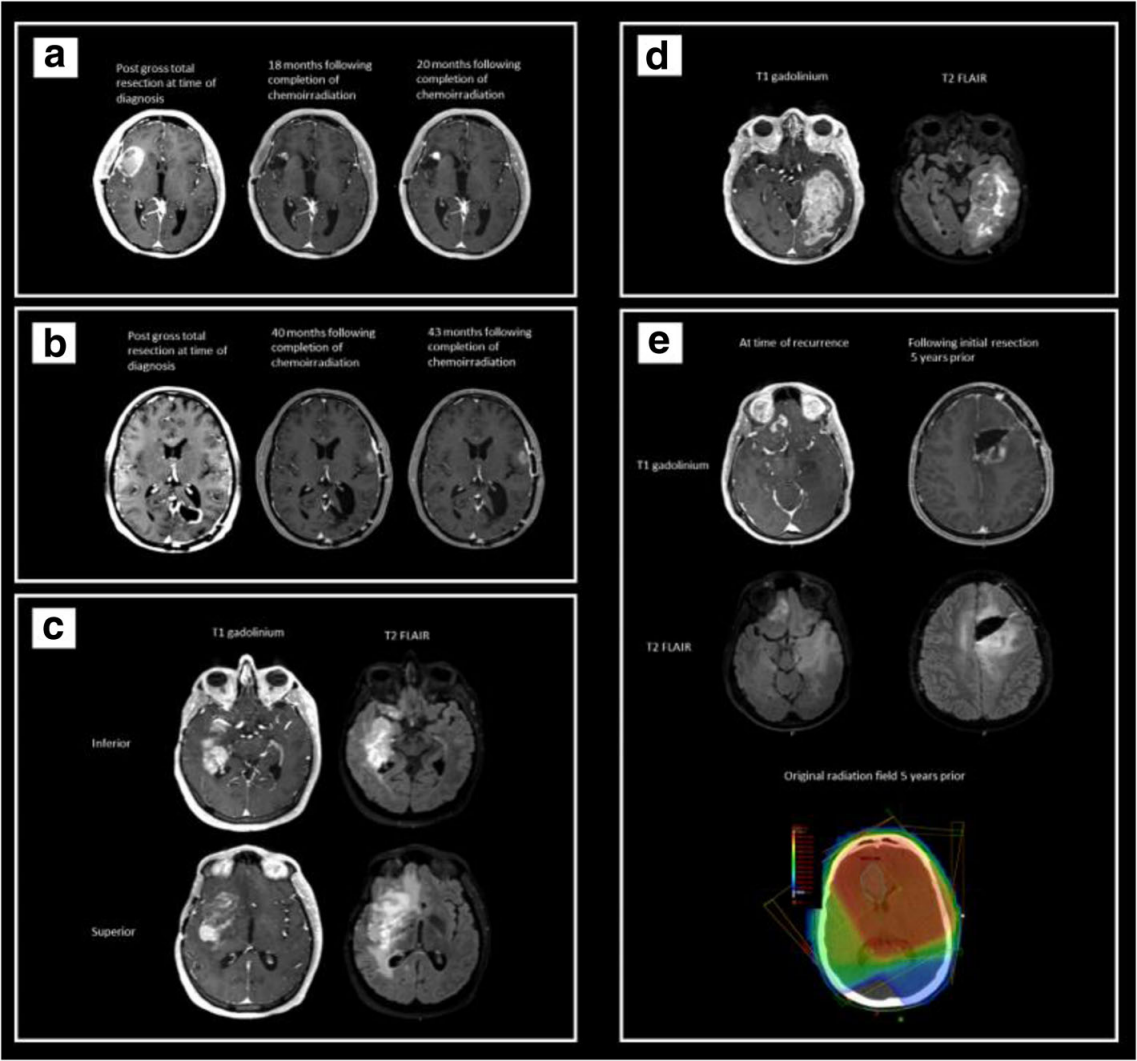
Imaging for the survey cases. **a** Case 1: a small in-field recurrence with a reasonably favorable location in a young patient who was 2 years out from chemoirradiation and had not been rechallenged with additional systemic agents. **b** Case 2: a small edge of field, biopsy proven recurrence in a reasonably favorable location in a younger patient almost 2 years out from treatment who had also failed Bevacizumab treatment. **c** Case 3: a larger in field recurrence, in an unfavorable location a considerable time out from treatment (11 years) who was heavily chemo-retreated, and had also failed Bevacizumab treatment. **d** Case 4: a very large in field recurrence in an older patient with a very good performance status who had completed chemoirradiation less than a year prior. **e** Case 5: a younger patient with an original histologic diagnosis of anaplastic astrocytoma who had done very well for 5 years until out of field progression was noted for which he was asymptomatic

This case involved a small in-field recurrence with a reasonably favorable location in a younger patient who was 2 years out from chemoirradiation and had not been rechallenged with additional systemic agents (Fig. 2a).

**Fig. 3:**
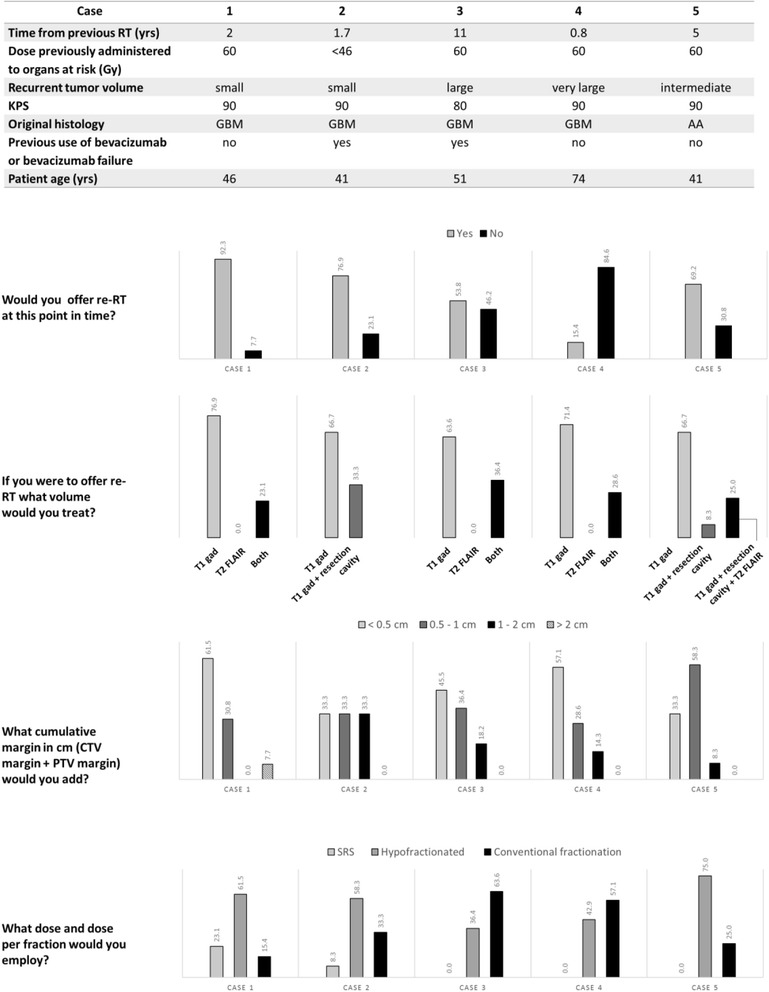
Re-irradiation case characteristics according to factors of relevance to offering re-RT as a treatment option and survey responses

All the responders considered offering re-RT to this patient (Fig. 3), though 1 responder did suggest seed implantation may be discussed as a possible next step. 77% of responders would treat the T1 gadolinium enhancing volume, with the remainder choosing to treat both the T1 gadolinium and the T2 FLAIR volume. Sixty two percent would employ a less than 0.5 cm cumulative margin and 31% would employ a margin ranging of 0.5 to 1 cm. Three responders would treat using stereotactic radiosurgery (SRS) techniques employing doses ranging from 18 to 25 Gy. The remainder of responders offered hypofractionated RT (62%), (30 to 43.2Gy in 5 to 18 fractions) or conventional fractionation (15%) (45–54 Gy in 25 to 30 fractions) (Fig. 3). Case 1 comments included: “dosing will depend on how much normal structures received in prior treatment; typically use 5 fractions or 10 if concerned with cumulative RT dose to normal structures” and “PRDR (pulsed reduced dose rate) is a technique that reduces the apparent rate at which the treatment is delivered to 6.67 cGy/min” as well as “Chemotherapy with Bevacizumab/CCNU” and “I would include PET-avid areas extending beyond the T1Gd MRI volume”. One responder summarized “Small focal recurrence at time of recurrence at almost 2 years post original treatment is not unreasonable to treat with SRS. Not growing quickly, though we do not have info on if FLAIR abnormality had progressed. if it has as well, I'd be more apt to treat FLAIR.”

#### Case 2

In contrast to case 1 this young patient had a biopsy proven recurrent glioblastoma outside of the 80% isodose line and also progressed on BEV (Fig. 2b).

In contrast to case 1, only 77% of responders considered offering re-RT to this patient, though 2 responder suggested resection as a possible next step. In comparison with case 1 the cumulative margin chosen in case 2 was more evenly divided among the options with 33%, 33%, 33% opting for less than 0.5 cm, 0.5 to 1 cm, 1 to 2 cm respectively, whilst in case 1 most responders had favored a smaller margin. 67% of responders would treat the T1 gadolinium enhancing volume, with the remainder choosing to treat both the T1 gadolinium plus the old resection cavity. 58%, 33%, 8% of responders offered hypofractionated re-RT (30 to 47.5 Gy in 5 to 19 fractions), conventionally fractionated re-RT (45 to 59.4 Gy) and SRS (18 Gy) respectively. This distribution was in contrast to case 1, where more responders chose to offer SRS or more extreme hypofractionation. As compared to case 1, in case 2, 42% responders decreased the dose per fraction they would employ while the remainder kept the same dose and fractionation in both cases (Fig. 3). Case 2 comments included: “discussion with the patient regarding stereotactic radiotherapy with single dose (18 Gy) or 6 * 5 Gy”, “SRS if no significant T2 signal, if there is a large area of T2 signal around the enhancement I would treat like the prior case”. With respect to BEV: “BEV causes the enhancement to underestimate the extent of the tumor. Moreover, this is an out of field failure. As such, I would treat to near full dose and continue the BEV” and another responder: “Treating only T1 increasing risk of marginal failure for patients progressing on BEV”. A responder summarized: “Large volume re-irradiation is justified given the long natural history.”

#### Case 3

In contrast to both case 1 and 2, this case involved a patient with glioblastoma who recurred 11 years following initial treatment, was heavily chemo-retreated and for whom all organs at risk in the field had previously received maximal dose (Fig. 2c).

Compared to case 1 or 2, only 54% of responders considered offering re-RT to this patient. Responders who chose to not offer re-RT did not do so as they felt that the patient would benefit more from a clinical trial (23%) or that the case was a palliative one with no role for re-RT (15%). Similarly, to case 1 and 2, 64% of responders would treat the T1 gadolinium enhancing volume, with the remainder choosing to treat both the T1 gadolinium and FLAIR volume. The cumulative margin chosen was 45%, 36%, 18%, for less than 0.5 cm, 0.5 to 1 cm, 1 to 2 cm respectively, more similar to case 1 where more responders favored a smaller margin but also reflecting the split in margin employed observed in case 2. In contrast to both case 1 and 2, only 33% of responders offered hypofractionated re-RT (30 to 35 Gy in 10 fractions), while 58% offered conventionally fractionated re-RT (36 to 54 Gy in 18 to 30 fractions) respectively. None of the responders offered SRS. Unlike either case 1 or 2, 2 responders also commented on continuing BEV and 2 others on the use of concurrent chemotherapy (CCNU or Temozolomide and BEV) (Fig. 3). Comments from the responders included: “After Avastin GTV/CTV/PTV not clear in MRI, MET-PET or FET-PET mandatory for RT decision”, “Consider concurrent CCNU or Temodar and BEV, TTF”. With respect to the OAR: “very high doses to the brainstem may be expected; should consider a rather low dose due to the size of the lesion” and “too big to hypofractionate. The FLAIR is clearly also failing so I would want to include. Use IMRT to constrain brainstem and optics. would want to continue the BEV on this case as well if possible.”

#### Case 4

Similar to case 3 this case involved a large recurrence volume with OAR having previously been treated to maximal dose however two features were in contrast to all other cases in this series in that this case involved an older patient (74 year old) who was out less than a year from previous treatment (Fig. 2d).

In contrast to all other cases, only 15% of responders considered offering re-RT to this patient, while the remainder 11 did not. Responders who chose to not offer re-RT did not do so as they felt that the recurrent tumor volume was too large for re-RT to be meaningful (31%) while the remainder felt that the patient would benefit more from a systemic option (54%). The responders were asked to provide a volume, margin and dose they would employ if they were to offer re-RT in this case even if they did not recommend it. Only 54% of responders did so. 71% of these would treat the gadolinium enhancing volume only, whereas 29% would treat both gadolinium enhancing volume and T2 FLAIR volume. The cumulative margin chosen was 57%, 29%, 14%, for less than 0.5 cm, 0.5 to 1 cm, 1 to 2 cm respectively. This bore the most similarity to the margin distribution in case 3. 3 and 4 responders offered hypofractionated re-RT (30 to 35 Gy in 10 fractions) and conventionally fractionated re-RT (36 to 54 Gy in 18 to 30 fractions) respectively, also similar to dose and fractionations offered in case 3. None of the responders offered SRS (Fig. 3). One responder included: “Wouldn't use re-irradiation at first failure this early for such a large recurrence. Patient may actually benefit from BEV symptomatically. clinical trial would be first option in my opinion.” Another responder stated “too large volume for a meaningful reirradiation” while another stated“chemotherapy with BEV and CCNU proposed”.

#### Case 5

In contrast to all the other cases, this case involved a patient with a recurrent anaplastic astrocytoma. It does share similarities otherwise to both case 1 and 2 as the patient is young and the recurrence is some distance away from the original resection (Fig. 2e).

Similarly, to Case 3, 69% of responders considered offering re-RT to this patient, while the remainder did not, although interestingly none of the responders who did so in case 3 overlap with case 5. Responders who chose to not offer re-RT did not do so preferring to recommend clinical trial (15%) or resection (15%). 67% of responders would treat the gadolinium enhancing volume alone, 8% would treat the gadolinium enhancing volume and the original resection cavity, 25% would treat the gadolinium enhancing volume, the original resection cavity and the T2 FLAIR volume. Similarly, to case 2, 31% responders would also re-RT the original resection cavity although only 15% of them overlapped with the two who did so in case 2. The cumulative margin chosen was 33%, 58%, 8%, for less than 0.5 cm, 0.5 to 1 cm, 1 to 2 cm respectively. 75% and 25% of responders offered hypofractionated re-RT (30 to 47.5 Gy in 10 to 19 fractions) and conventionally fractionated re-RT (36 to 54 Gy in 18 to 27 fractions) respectively. None of the responders offered SRS (Fig. 3). Five Comments from 5 different responders emerged: “five years is a long time, I would be aggressive”, “I would always recommend concurrent BEV with re-irradiation; that goes for all the cases I mentioned before where I would re-treat.”, “T2 signal goes around the chiasm. Feel better not treating the T2 if additional systemic treatment is added” and “Would prefer sampling of new and old site. If prior site consistent with treatment changes only, then treat new foci only”. With respect to OAR one responder summarized as follows: “Limited volume out of field recurrence. Optic tolerance will play a role in re-irradiation planning. Because of large volume previously irradiated, I would want to just treat the recurrent region and probably shouldn't go to full dose given that the optics probably previously maxed out.”

## Discussion

Re-irradiation is being offered for patients with recurrent glioma at both academic centers and in the community, on clinical protocols or outside of existing protocols. From retrospective data [2–12] we understand that re-RT is feasible and that toxicity, if it occurs does not appear to be significant or clinically relevant. Despite numerous publications on re-RT in patients with recurrent glioma and several active protocols, we noted that there was no publication that specifically discussed the approach experts would adopt when faced with a potential re-RT case especially outside of a study protocol. Specifically, the question of our survey rested with the treatment volume, margins, dose and fractionation and more so than patient selection (seeing as this an evolving topic in and of itself with several available scoring systems [14–18]. It is this information that we are frequently consulted on as re-RT experts and therefore, we felt that it was important to the recurrent glioma re-RT field to publish an approach other providers could follow and to provide a platform that others could contribute to (the live survey link below) in order to advance the field. To our knowledge this is in the only such survey and the only paper to discuss volumes, margins, dose and fractionation with respect to re-RT.

We obtained the opinion of radiation oncologists who share an interest in re-RT of patients with recurrent glioma using a simple survey involving 5 re-RT cases that could represent more common scenarios encountered in clinic as starting point for discussion and development of consensus. The responders share some common features including 1) all except one have treated more than 20 patients, although 3 of them have treated more than a 100, 2) they see at least 2 patients referred for re-RT per month and 3) treat at least 1 patient per month. With the data presented here on 13 responders, the numbers were insufficient to draw any conclusions with respect to difference in approach to patient selection or management based on the level of expertise of the responders although it is likely that such trends may emerge once the paper is published, readers take the survey and more data is available. We are aware of re-irradiation being practiced in the community as well as in academic centers and this too will be interesting to analyse. The volume seen in the community is likely far smaller than what the experts in the our paper have, which in a sense makes ours more homogenous of a cohort. Patient selection criteria, dose and fractionation to be employed are heterogeneous. Equally so is the perceived impact of previously administered dose to OAR with most responders rating it as extremely or very important while proceeding to re-RT with doses that are often unlikely to respect dose constraints. Despite the dose to the OAR having been reached dose limit in the first radiation course in all but one patient, greater than 50% of responders elected to offer re-RT in all but one case in which per responders’ comments, the reason for not offering re-RT was tumor size as opposed to dose to OAR. Perhaps this reflects a belief in normal tissue repair with increasing time from previous RT or a belief in lower dose per fraction decreasing the risk of late toxicity. These two considerations could be reflected in the minimal use of SRS (case 1 and 2).

Small volume “in field” recurrence in a young patient with a disease free interval of 2 years (case 1) prompted close agreement among responders all of whom perceived this case as an opportunity to elicit local control for the patient while considering both the risk of possible toxicity and the potential for longevity. Most responders chose a smaller margin and some employed SRS, although clearly hypofractionation was still favored by most responders irrespective of their geographical practice location. Three responders did choose to include T2 FLAIR and/or FDG-PET avid areas extending beyond the T1 gadolinium volume and some also pointed out that they would ultimately decide on their dose per fraction depending on dose previously administered to OAR. Offering clinical trials as well as considering concurrent chemotherapy with BEV and/or Lomustine was also mentioned.

The “out of field” BEV failure case (case 2) prompted the use of larger margins with concern for more extensive occult disease presence due to previous treatment with BEV. There was increased use of hypofractionation and conventional fractionation as opposed to SRS. Even when single fraction SRS was mentioned, the responders felt that hypofractionation should be considered and discussed with the patient. Several responders commented on this case representing an out of field recurrence, hence the willingness and ability to treat to full dose but also the concern that treatment of the T1 gadolinium enhancing tumor without addressing the T2 FLAIR or employing a larger margin, could result in marginal failure. In keeping with this view, 4 responders also chose to include the original resection cavity despite this being an out of field recurrence where the original resection cavity had remained stable. This too caused responders to favor hypofractionation or conventional fractionation over single fraction SRS.

A large volume recurrence with maximal previous dose to OAR (case 3), caused almost half the responders to not offer re-RT favoring either clinical trial or best supportive care. Responders also commented that re-RT could only be recommended if the OAR could be spared. Of the responders who did offer re-RT some rationalized that the long natural history and superior KPS did justify large volume re-RT but also stated that they would avoid hypofractionation.

In the older patient with a disease interval of less than a year from the original radiation (case 4) most responders would not offer re-RT, favoring systemic treatment or other options. The options included BEV plus CCNU, BEV alone or possible tumor treating fields (TTF). The responders who did offer re-RT in this case did so while expressing the concern of this representing possible pseudoprogression and the need to rule this out.

The case of recurrent anaplastic astrocytoma (case 5) was more split between resection or clinical trial and re-RT. Some of those who would re-RT, commented that they would consider a biopsy of the original cavity to help decide as to whether they would include it in their volume vs. treating the new enhancement alone. Some expressed that this was a limited volume out of field recurrence where optic nerve tolerance would play a role in re-RT planning and therefore they would treat the recurrent region only, given that the optic apparatus had previously received maximal dose. Two responders stated that they would treat with concurrent BEV and another that they would treat aggressively considering the long interval from previous RT. Some commented on foregoing treatment of the T2 FLAIR signal area as enveloping the chiasm and would especially consider doing so if systemic concurrent treatment were given. This case reflected the most heterogeneity in terms of volume to be treated and the use of systemic treatment as well as underlying rationale.

The question of clinical benefit as well as the concern over toxicity are not being addressed in this paper and remain a matter of debate in the absence of robust randomized trials that carry a best supportive care arm. Re-RT remains therefore remains controversial. We acknowledge that many patients are not referred for re-RT due to real or perceived lack of clinical benefit on the part of the provider. Therefore, the patients who do obtain re-RT reflect some level of selection bias. Cases attempting to elucidate the perception of clinical benefit on the part of the provider and hence the decision to offer re-RT were deliberately included in the questionnaire (especially so Case 3 and 4). We also acknowledge that this paper represents a reflection of the current practice in a number of centers who do practice re-RT. Prospective evidence is lacking.

Overriding patterns of practice/recommendations for management:

1. Offer re-RT to patients with smaller recurrences, especially if:

- located in a favorable location.
- ability to spare or minimize dose to OAR.
- long interval since previous RT defined as greater than or equal to 6 months.
- well defined area of recurrence ie. no previous use of BEV/BEV failure.
- consider SRS or hypofractionated dose/fractionation in cases that meet size and location criteria above.

2 Consider clinical trial, systemic treatment or best supportive care in cases with:

- Large volume recurrence.
- Short interval since previous RT defined as less than 6 months.
- unclear clinical and radiographic progression in the absence of tissue confirmation.

3 Consider re-RT on a case by case basis in scenarios where:

- OAR have received maximal dose previously and cannot be spared if further RT given.
- Surgical resection possible.
- If re-RT is proposed in cases where OAR toxicity is a concern, most responders would favor conventional fractionation over hypofractionation.

The lack of published toxicity following re-RT in the setting of doses that exceed published dose constraints reflect the limited life expectancy of patients treated as well as perhaps a significant tumor related neurologic deterioration which prevents adequate attribution of toxicity following re-RT. The authors recommend rigorous testing of visual fields, audiology, neurocognitive function prior to and following re-RT to better examine possible impact on toxicity as well as patient reported outcomes for quality of life metrics.

The cumulative dose administered to OAR as a result of re-RT and therefore the impact of those doses remains unclear. The limited toxicity observed should be examined further as per testing above but also should prompt the collection of OAR doses among the RT treatments for the purposes of designing a model of toxicity risk. Current guidelines [19, 20] lack the data to make recommendations or offer risk estimation for doses above 60 Gy or with hypofractionated regimens.

The following were not addressed in this study and require further research: the most optimal timing of re-RT, the co-administration of systemic agents and the impact of re-RT on brain imaging specifically the interpretation of treatment failure versus tumor progression on MRI.

Seeing as the process underlying the decision to offer re-RT is more complex than the patient or disease specific factors deemed relevant in the questionnaire, we suspect that the decision to offer re-RT is likely based on a collection of case features as summarized in the “Overriding patterns of practice/recommendations for management” section. The need to understand this better in a larger sample is part of the rationale for the inclusion of a live link available for the duration of a year following publication. This survey will be available as live link (https://www.surveymonkey.com/r/G7MWVZ8) for radiation oncologists willing to take it for one year from publication and results thereof will be published once available.

## Conclusions

Data for the optimal administration of re-RT is lacking but some overriding concepts emerged in this survey governing the administration of re-RT within and outside of a clinical trial as evidenced by the authors. Existing prognostic scores need to be further refined to reflect the outcome of patients with recurrent glioma who undergo re-RT. Ultimately it appears that patient selection is predicated on time since previous RT, previously administered dose and tumor volume, some of which are not currently addressed in scoring systems. Ongoing collaboration and focus on patient selection for re-RT will continue to enable the development of scoring systems that reflect patient outcome, provider clinical impression and radiobiology principles.

## Additional file

Additional file 1: Survey Monkey. (PDF 2056 kb)

## Abbreviations

BEV: Bevacizumab
CCNU: Lomustine
CNS: Central Nervous System
DICOM: Digital Imaging and Communications in Medicine
DVH: Dose Volume Histogram
FLAIR: Fluid attenuation inversion recovery
FSRT: Fractionated Stereotactic Radiotherapy
IDH: Isocitrate Dehydrogenase
IMRT: Intensity Modulated Radiation Therapy
KPS: Karnofsky Performance Status
MGMT: O6-methylguanine–DNA methyltransferase
MRI: Magnetic Resonance Imaging
NCI: National Cancer Institute
OAR: Organs at Risk in the Field
PET: Positron emission tomography
PRDR: Pulsed Reduced Dose Rate
Re-RT: Re-irradiation
RT: Radiation Therapy
SRS: Stereotactic Radiosurgery
TMZ: Temozolomide
TTF: Tumor Treating Fields
