# Correction to expert consensus on re-irradiation for recurrent glioma

**DOI:** 10.1186/s13014-018-0955-8

**Published:** 2018-01-18

**Authors:** Andra V. Krauze, Albert Attia, Steve Braunstein, Michael Chan, Stephanie E. Combs, Rainer Fietkau, John Fiveash, John Flickinger, Anca Grosu, Steven Howard, Carsten Nieder, Maximilian Niyazi, Lindsay Rowe, Dee Dee Smart, Christina Tsien, Kevin Camphausen

**Affiliations:** 1 Radiation Oncology Branch, National Cancer Institute NIH, Bethesda, MD USA; 2 Department of Radiation Oncology, Vanderbilt University Medical Center, Nashville, TN USA; 3 Department of Radiation Oncology, University of California, San Francisco, USA; 4 Department of Radiation Oncology, Wake Forest Baptist Medical Center, Winston-Salem, NC USA; 5 Department of Radiation Oncology, Technical University of Munich (TUM), Munich, Germany; 6 Institute of Innovative Radiotherapy (iRT), Department of Radiation Sciences (DRS), Helmholtz Zentrum München, Neuherberg, Germany; 7 Deutsches Konsortium für Translationale Krebsforschung (DKTK) Partner Size, Munich, Germany; 8 Department of Radiotherapy Erlangen, Erlangen, Germany; 9 Department of Radiation Oncology, University of Alabama at Birmingham, Birmingham, AL USA; 10 UPMC Presbyterian-Shadyside, Pittsburgh, PA USA; 11 Department of Radiation Oncology, University of Freiburg, Freiburg, Germany; 12 Department of Human Oncology, University of Wisconsin, Madison, WI USA; 13 Department of Oncology and Palliative Medicine, Hospital Trust, 8092 Bodø, Nordland Norway; 14 Department of Clinical Medicine, Faculty of Health Sciences, University of Tromsø, 9038 Tromsø, Norway; 15 Department of Radiation Oncology, LMU Munich, Marchioninistr. 15, 81377 Munich, Germany; 16 German Cancer Consortium (DKTK), German Cancer Research Center (DKFZ), Heidelberg, Germany; 17 Radiation Oncology Branch, National Cancer Institute, NIH, Bethesda, MD USA; 18 Department of Radiation Oncology, Washington University, St. Louis, MO USA

## Correction

In the original publication [1] two author names were missing the middle names. The corrected versions can be found in this Erratum.

The original article was updated to rectify these errors.

Incorrect author name 1:

Stephanie Combs.

Correct author name 1:

Stephanie E. Combs.

Incorrect author name 2:

Andra Krauze.

Correct author name 2:

Andra V. Krauze.
