# Supplementary material for: Expert consensus on re-irradiation for recurrent glioma
Source: Radiat Oncol. 2017 Dec 1;12:194. doi: 10.1186/s13014-017-0928-3 (PMC5709844; doi:10.1186/s13014-017-0928-3)
Supplement: Additional file 1: — Survey Monkey. (PDF 2056 kb) [file 13014_2017_928_MOESM1_ESM.pdf]

Expert consensus on re-irradiation for recurrent glioma

Re-irradiation in patients with recurrent glioma

Re-irradiation in patients with recurrent glioma has been shown to be feasible in a number of retrospective studies. Re-irradiation may offer palliation of symptoms and may in some patients also improve outcome. Guidelines governing patient selection for re-irradiation as well as the volume, dose and fractionation to be employed are lacking. While trials examining these questions are ongoing, many patients may not meet trial criteria and may nonetheless receive re-irradiation when no other options are available.

This survey will form the backbone of an expert consensus paper on this subject and you are kindly invited to participate. The survey will be distributed to a very limited number of radiation oncologists who treat central nervous system tumors and have a specific interest in re-irradiation of glioma. The survey is divided into 2 sections.

The first section containing 10 questions is a demographic section and will help assess the load of re-irradiation cases you treat/have treated and ensure that your contact is available such that you may be included on the final publication.

The second and final section contains 5 re-irradiation cases with 5 to 6 questions associated with each case.

A comment section is available following each case. Any comments you may have with respect to the cases or the questions themselves would be very welcome and will help strengthen the understanding of how radiation oncologists approach these challenging scenarios and ultimately decide on management.

Thank you in advance for your opinion and for taking the time to answer these questions.

Responder demographics

1. What is your name?

2. Which institution are you currently working at?

3. How many years have you been practicing radiation oncology?

- < 5 years
- 5 - 10 years
- > 10 years

4. How many patients with recurrent glioma have you re-irradiated to date?

- < 20
- 20-100
- 100-200
- > 200

5. How many potential glioma re-irradiation cases are you seeing per month?

- 1-2
- 2-5
- 5-10
- > 10

6. How many patients with recurrent glioma do you re-irradiate per month?

- 1-2
- 2-5
- 5-10
- > 10

7. Are the patients with recurrent glioma who receive re-irradiation being treated on a clinical study?

- ◯ yes, all patients are being re-irradiated on study
- ◯ some patients are being re-irradiated on study if they meet study criteria, while others are treated off study
- ◯ all patients are treated off-study

8. Re-irradiation is being offered with increasing frequency to patients with recurrent glioma, however evidence governing patient selection, dose/fractionation, volume to be treated and the administration of concurrent agents is lacking. On a scale from 1 (not at all relevant) to 4 (extremely relevant), please rate how relevant each factor is to your decision to offer re-irradiation as a treatment option in patients with recurrent glioma.

	Not at all relevant	Somewhat relevant	Very relevant	Extremely relevant (crucial for decision making)	
Tumor volume	◯	◯	◯	◯	◯
Time since previous radiation therapy	◯	◯	◯	◯	◯
Dose previously administered to organs at risk in the field	◯	◯	◯	◯	◯
Original histology	◯	◯	◯	◯	◯
Patient age	◯	◯	◯	◯	◯
Patient performance status	◯	◯	◯	◯	◯
Number of lines of previous treatment	◯	◯	◯	◯	◯
Previous use of Bevacizumab or Bevacizumab failure	◯	◯	◯	◯	◯
Available tissue documentation of tumor progression	◯	◯	◯	◯	◯
Perfusion characteristics on Diffusion Weighted Imaging	◯	◯	◯	◯	◯
PET avidity of the lesion	◯	◯	◯	◯	◯

9. Based on personal experience, please mention any additional factors you consider relevant when considering re-irradiation in a patient with recurrent glioma.

[text box]

10. Do you routinely use any of the following measures in making a decision regarding offering re-irradiation as treatment option or as part of the treatment planning process? (Yes/No)

	Yes	No
Multidisciplinary discussion with medical/neurooncology/neurosurgery	○	○
Obtaining previous radiation therapy treatment summary and plan information (DVH, field arrangement)	○	○
Obtaining previous treatment plan in DICOM format to create a plan sum	○	○
Calculate cumulative dose to organs at risk in the field taking into account multiple treatment plans	○	○

Re-irradiation cases - Case 1.

Thank you for participating in our survey. Your feedback is important.

46 year old female with glioblastoma, MGMT methylated, post gross total resection, concurrent chemoirradiation and 6 cycles of adjuvant Temozolomide with suspected radiographic tumor recurrence 2 years following completion of treatment. Patient is refusing repeat surgical intervention. KPS 90. The patient is asymptomatic, the lesion at the edge of the resection cavity is perfused on DWI and PET avid.

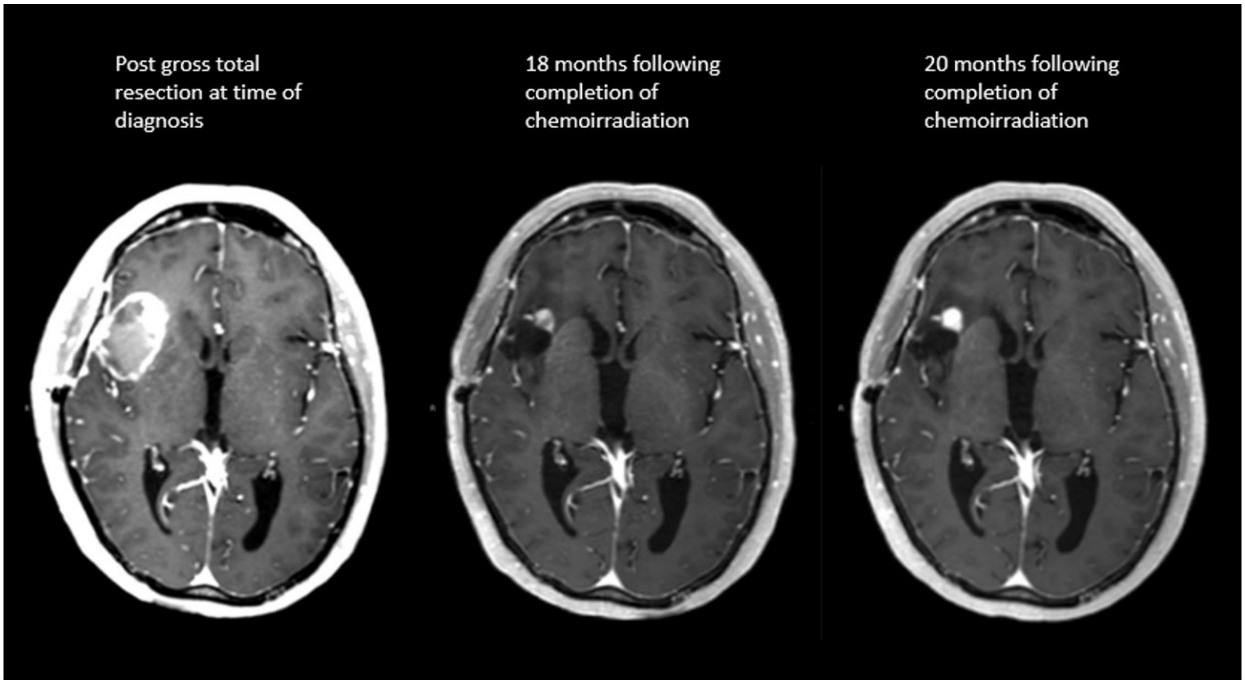

11. Would you offer re-irradiation as a treatment option at this time?

If not, what next treatment option would you propose?

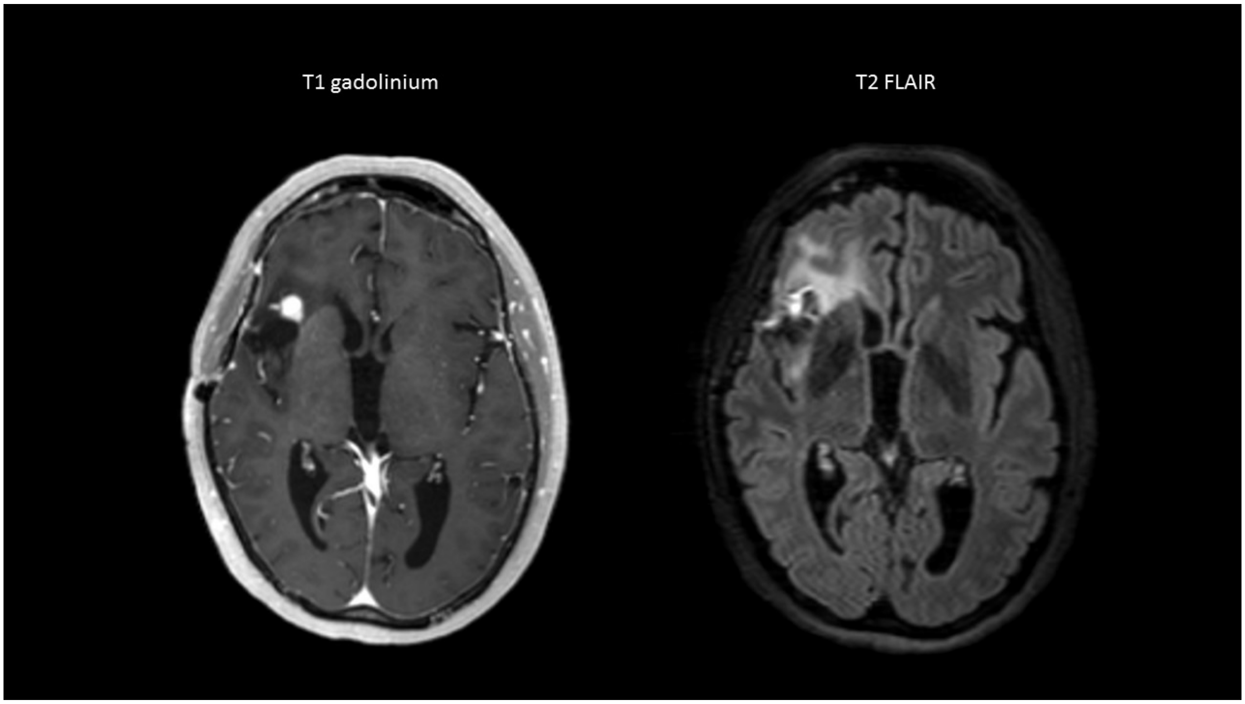

12. If you were to offer re-irradiation, what volume would you treat?

13. What cumulative margin (CTV margin + PTV margin) would you add?

14. What technique would you employ?

15. What dose would you employ (cGy)?

16. What dose per fraction would you employ (cGy)?

17. Additional comments

Re-irradiation cases - Case 2.

41 year old female with diagnosis of glioblastoma treated with grosstotal resection followed by concurrent chemoirradiation and 20 cycles of adjuvant temozolomide, with radiographic progression and initiation of Bevacizumab followed by further radiographic progression with an enlarging T1 gadolinium enhancing lesion and no associated T2 FLAIR changes. A biopsy of the lesion revealed glioblastoma with gliosarcomatous differentiation.KPS 90.

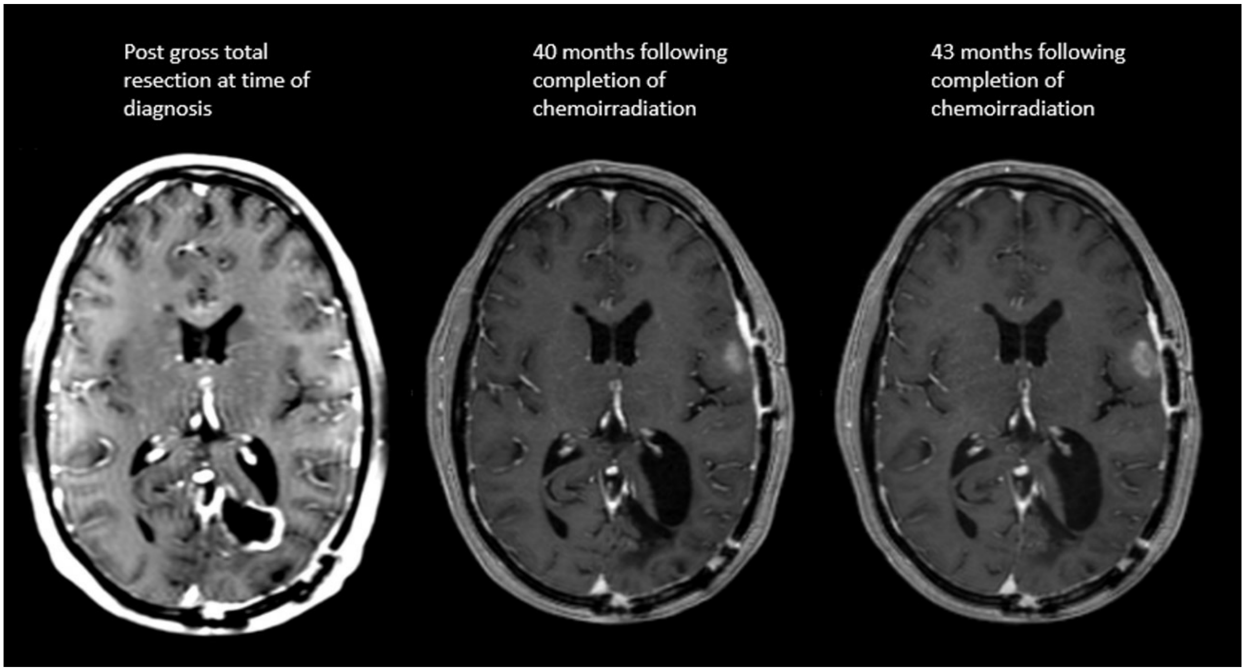

18. Would you offer re-irradiation as a treatment option at this point in time?

If not, what next treatment option would you propose?

19. If you were to offer re-irradiation, what volume would you treat?

Other (please specify)

20. What cumulative margin (CTV margin + PTV margin) would you add?

21. What treatment technique would you employ?

22. What dose would you employ (cGy)?

23. What dose per fraction would you employ (cGy)?

24. Additional comments

Re-irradiation cases - Case 3.

51 year old male, glioblastoma diagnosed 11 years ago, treated with gross total resection, followed by concurrent chemoirradiation to 60 Gy in 30 fractions and subsequently 24 cycles of temozolomide, with multiple recurrences treated with repeat resection, temozolomide, CCNU/avastin and everolimus/sorafenib. KPS 80. Clinical and radiographic progression. All organs at risk in the field have previously been treated to maximal dose.

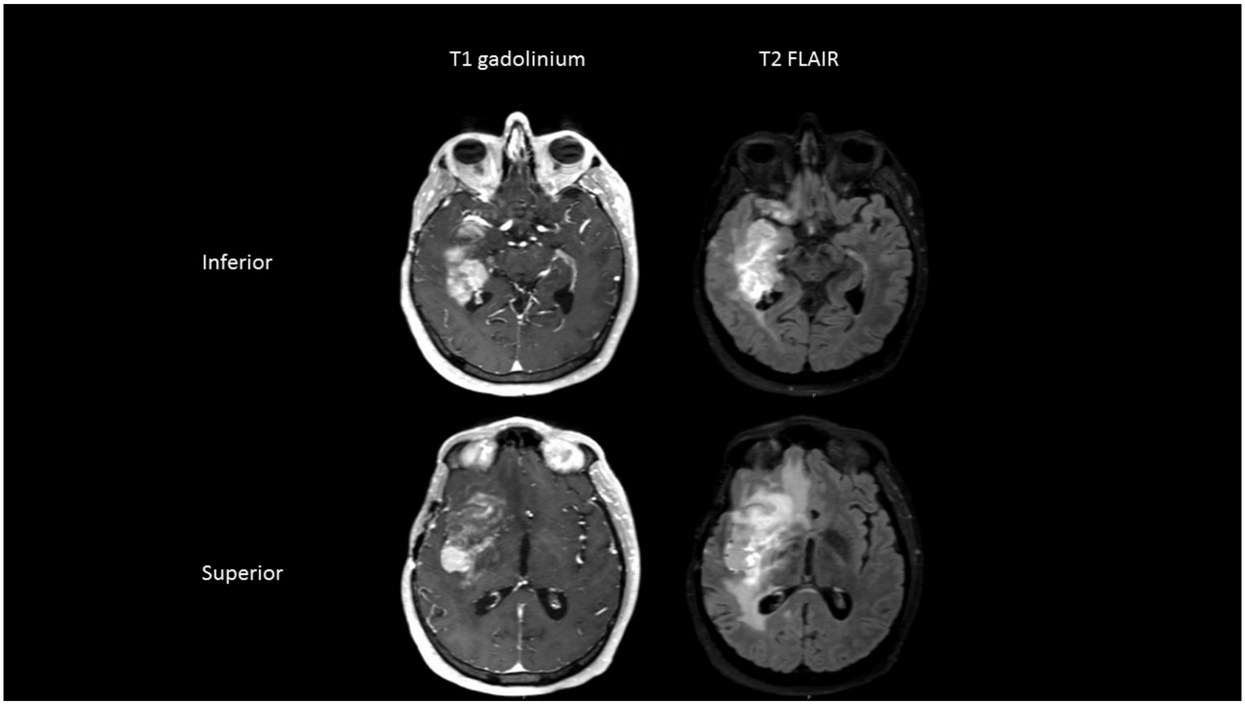

25. Would you offer re-irradiation as a treatment option at this point?

If not, what next treatment option would you propose?

26. If you were to offer re-irradiation, what volume would you treat?

Other (please specify)

27. What cumulative margin (CTV margin + PTV margin) would you add?

28. What dose would you employ? (cGy)

29. What dose per fraction would you employ?

30. Additional comments

Re-irradiation cases - Case 4

74 year old male diagnosis of glioblastoma treated with standard chemoirradiation finishing 10 months ago, post adjuvant temozolomide with radiographic recurrence, clinically mildly symptomatic. KPS 90.The recurrence is entirely within the previous treatment field and all organs at risk in the field received maximal dose.

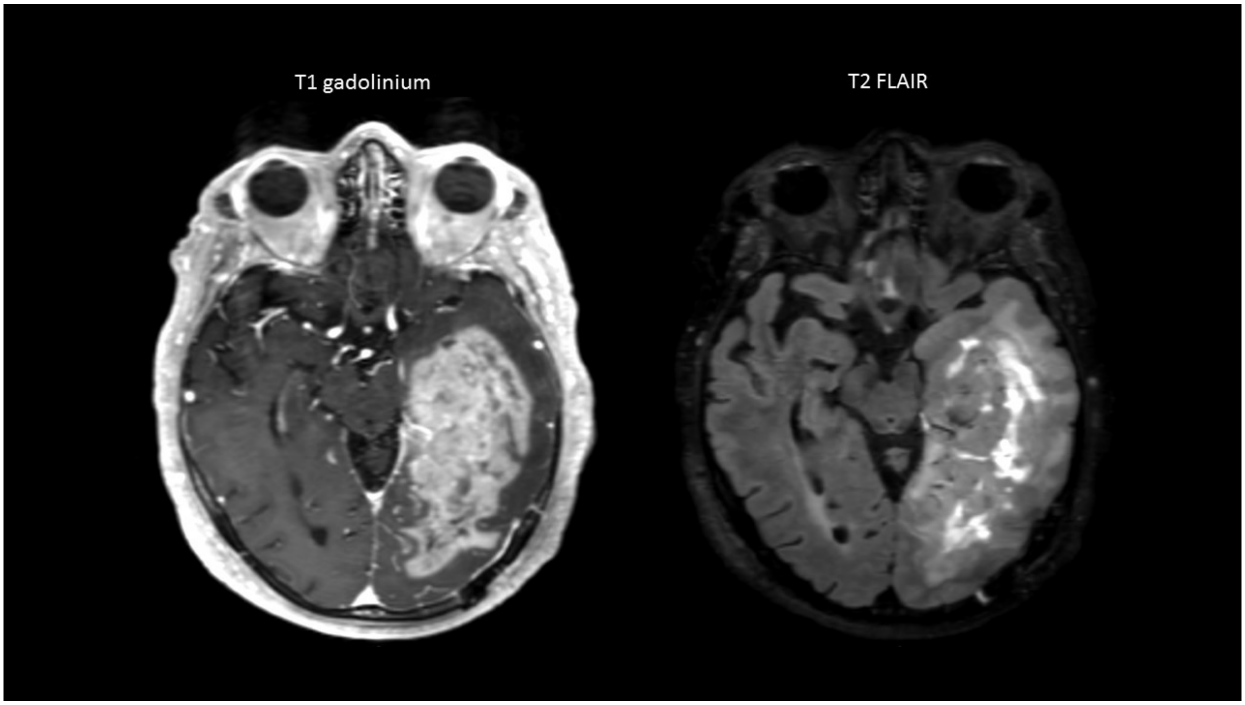

31. Would you offer re-irradiation as a treatment option at this point?

If not, what next treatment option would you propose?

32. If you were to offer re-irradiation, what volume would you treat?

Other (please specify)

33. What cumulative margin (CTV margin + PTV margin) would you add?

34. What dose would you employ (cGy)?

35. What dose per fraction would you employ (cGy)?

36. Additional comments

Re-irradiation cases - Case 5.

41 year old male with diagnosis of anaplastic astrocytoma treated with concurrent chemoirradiation 5 years prior, with radiographic progression, clinically asymptomatic. Following rechallenge with temozolomide, there is ongoing progression. KPS 90.

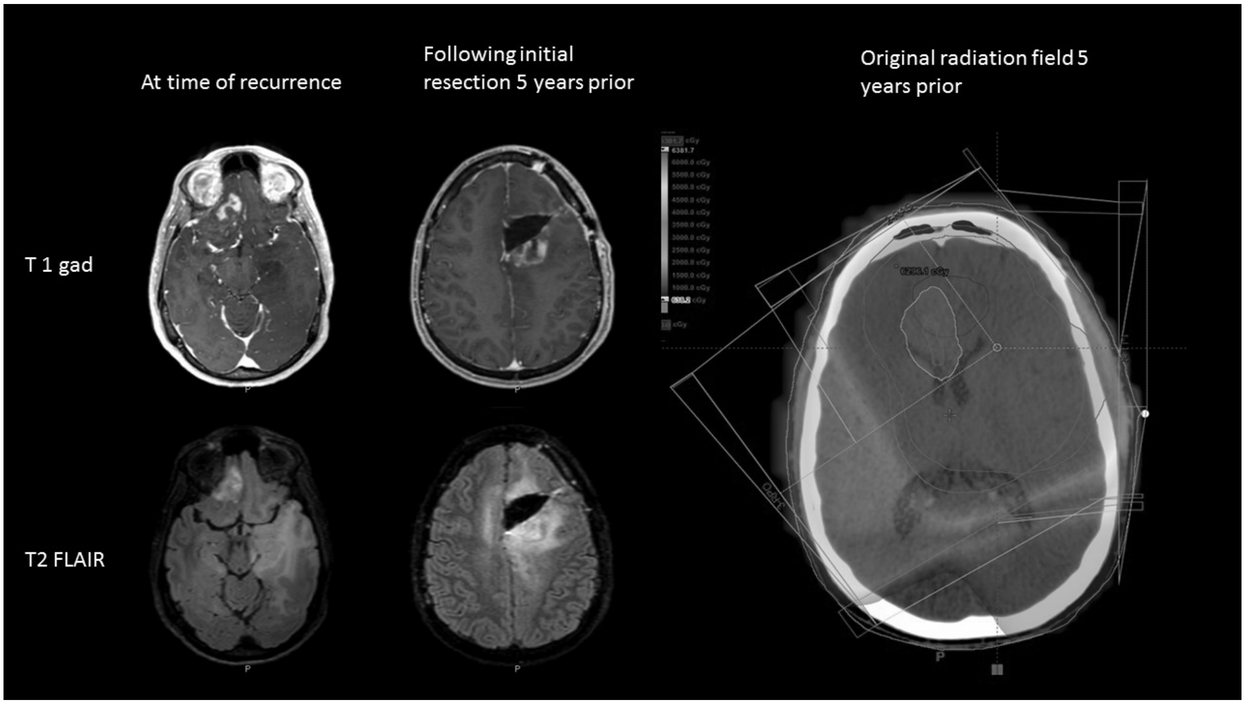

37. Would you offer re-irradiation at this point in time?

If not, what next treatment option would you propose?

38. If you were to offer re-irradiation, what volume would you treat?

Other (please specify)

39. What cumulative margin would you add (CTV margin + PTV margin)?

40. What dose would you employ (cGy)?

41. What dose per fraction would you employ (cGy)?

42. Would you consider the administration of a concurrent systemic agent?

43. Additional comments
